# Phenotypic, Genetic and Environmental Architecture of the Components of Sleep Quality

**DOI:** 10.1007/s10519-022-10111-0

**Published:** 2022-08-25

**Authors:** Juan J. Madrid-Valero, Juan F. Sánchez-Romera, Jose M. Martínez-Selva, Juan R. Ordoñana

**Affiliations:** 1 Department of Health Psychology, Faculty of Health Sciences, University of Alicante, 03690 Alicante, Spain; 2 Department of Human Anatomy and Psychobiology, University of Murcia, Campus de Espinardo, 30100 Murcia, Spain; 3 Murcia Institute of Biomedical Research, IMIB-Arrixaca, 30120 Murcia, Spain

**Keywords:** Dizygotic, Monozygotic, Pittsburgh sleep quality index, Sleep quality, Twins

## Abstract

The genetic and environmental underpinnings of sleep quality have been widely investigated. However, less is known about the etiology of the different sleep quality components and their associations. Subjective sleep quality has been studied most commonly using the Pittsburgh Sleep Quality Index (PSQI). Therefore, this work aimed to study the structure of sleep quality dimensions in a population-based twin sample by examining the etiology of the associations among the PSQI components themselves and between them. The sample comprised 2129 participants from the Murcia Twin Registry. In order to study the phenotypic, genetic and environmental structure of the PSQI we used three alternative multivariate twin models including all seven sub-scales of the PSQI (subjective sleep quality, latency, duration, efficiency, disturbances, use of sleeping medication and daytime dysfunction): a multivariate model (with seven separate correlated factors), a common pathway model and an independent pathway model. The multivariate correlated factors model showed the best fit to the data. All twin models indicated significant genetic overlap among most of the PSQI components, except daytime dysfunction and use of sleep medication. Bivariate heritability explained between 25 and 50% of the covariance for most associations between dimensions. Furthermore, the common pathway model showed that around one third of the variance (0.32; CI 95% 0.18.0.43) of a latent factor common to all questionnaire dimensions is explained by genetic factors. Genetic influences on a latent factor common to all questionnaire dimensions produced the same heritability estimates as the PSQI global score. However, sleep quality dimensions showed considerable specificity regarding its genetic-environmental structure.

**Supplementary Information:**

The online version contains supplementary material available at 10.1007/s10519-022-10111-0.

## Introduction

Sleep quality is a broad concept encompassing different aspects of sleep and is widely used in both research and clinical practice (Krystal and Edinger 2008). Although sleep quality has been measured using different strategies, from physiological responses to a single-item self-report question, there is some consensus that it encompasses several complementary aspects such as sleep duration, latency, efficiency, degree of fragmentation, total wake time, sleep disruptive events and daytime dysfunction among others (Krystal and Edinger 2008). From this comprehensive perspective, the Pittsburgh Sleep Quality Index (PSQI) (Buysse et al. 1989) is the most widely used questionnaire and has proven to be a useful, valid and reliable measure of sleep quality, and deemed by some researchers as the gold standard for subjective sleep quality (Carpenter and Andrykowski 1998; Boudebesse et al. 2014). Specifically the PSQI assesses seven components of sleep quality: (1) subjective sleep quality (subjective perception of sleep quality); (2) sleep latency (amount of time it takes to go from wakefulness to sleep; (3) sleep duration (total amount of sleep); (4) sleep efficiency (ratio between sleep duration and the total time dedicated to sleep, normally given as a percentage); (5) sleep disturbances (frequency of alterations during sleep such as: cough, snoring, heat or cold among others); (6) use of sleeping medication (frequency of sleeping medication use); (7) daytime dysfunction (sleepiness and fatigue while doing daily activities).

Different studies have supported the unifactorial structure of this questionnaire (Manzar et al. 2016; Raniti et al. 2018) and phenotypic correlations between PSQI dimensions are usually significant. However, they do not overlap completely, and between-component associations may vary widely [e.g., from 0.07 to 0.64 (Raniti et al. 2018)]. Additionally, each dimension may show distinct relationships with other variables [e. g., sleep duration and obesity (Cappuccio et al. 2008) or use of sleeping pills with anxiety (Harris et al. 2018)]. In summary, there is substantial heterogeneity between sleep quality components and the etiology of their associations is not fully understood yet. Therefore, advancing knowledge about the psychometric structure of this construct, the etiology of each component of sleep quality and how they relate to each other puts us in a stronger position to design and develop more accurate and effective treatments and prevention programs since sleep is a potential modifiable determinant of health and well-being (Espie et al. 2019).

Several studies have addressed the genetic and environmental influences on sleep quality and specifically the PSQI. For example, in a recent meta-analysis of twin studies, eight publications for sleep quality were included, five of which and the most recent used the PSQI to measure sleep quality (Madrid-Valero et al. 2020). Heritability estimates for the global PSQI index are around 0.30–0.50 (Madrid-Valero et al. 2020; Kocevska et al. 2021). Yet, less is known about the genetic and environmental structure of the PSQI components and their relationships. Few studies have estimated the heritability of the PSQI components, reporting a range from 0.20 to 0.45 (Barclay et al. 2010a; Madrid-Valero et al. 2018; Genderson et al. 2013). For example, Barclay et al. (2010a) reported heritability estimates from 0.21 to 0.47, in a sample of young adults, for all components except sleep duration, which showed no evidence of genetic factors, and use of sleep medication, which was not analyzed due to the age of the sample. Genderson et al. (2013) also estimated the heritability of the PSQI components using a sample of middle-aged men. They found heritability estimates ranging from 0.23 to 0.34 except for use of use of sleeping medication, where no genetic influences were detected. Finally, Madrid-Valero et al. (2018) using a sample of middle-aged men and women from Spain, found heritability estimates between 0.30 to 0.45, except for habitual sleep efficiency.

The above cited literature highlights the role of genetic factors in explaining individual differences in sleep quality and their components. However, there remain discrepancies regarding the magnitude of such influences—which are likely due, at least in part to the different characteristics of the studied samples—and doubts about the nature of the associations between the components of sleep quality. Furthermore, structures other than originally proposed by Buysse et al. (1989) have been suggested. Thus, Cole et al. (2006) postulated a 3-factor model with 3 clusters named sleep efficiency, perceived sleep quality and daily disturbances.

Despite these uncertainties just one study has addressed the etiology of the relationships between PSQI dimensions. Barclay et al. (2010a) suggested that the associations between components may be influenced differently by genetic factors, and their examination could help to gain a more thorough understanding of the constructs encompassed by the PSQI. By analyzing the relationships between components using a series of bivariate twin models, they claimed partial support for the proposed 3-factor structure as some of the paired correlations were particularly strong between pairs of components clustered in the same factor (e.g., subjective sleep quality and sleep latency or habitual sleep efficiency and sleep duration). However, they did not actually model the factorial structure and, furthermore, many of the other relationships did not fit properly into the three-factor architecture.

Interesting as it is, being the only one analyzing this question, Barclay et al.’s study presents some aspects that could be ameliorated. They used a pair-wise approach which only provides a mosaic of partial pictures of the PSQI etiological structure, not properly taking into account the possible effect of the other components on each paired association. Moreover, their study did not have enough power to accurately detect the etiological correlations between components, as shown by the very wide confidence intervals for some of those estimates.

Nevertheless, acquiring precise knowledge on the nature and characteristics of the associations among the sleep quality components is highly relevant for a variety of reasons: it might offer a possible explanation on why some components tend to cluster together; provide a deeper insight into the psychometric structure of the PSQI; reveal a more detailed characterization of sleep problems and enable better identification of individuals at risk (for example if a high genetic overlap is confirmed, this would suggest that patients with problems in one of the sleep quality dimensions could also be genetically sensitive to correlated symptoms); serve as a framework and guidance for gene identification efforts; and, of course, facilitate a more thorough understanding of the very concept of sleep quality and its associated problems.

Twin research methods enable the development of a wide variety of designs that could shed further light on the etiology of the relationship among the sleep quality components. In this regard, while a multivariate model allows for the estimation of the genetic and environmental influences on both individual variance and sources of covariance among the seven PSQI subscales, a common pathway model would serve to explore the possibility of genetic and environmental factors influencing each of the seven PSQI subscales via a common latent factor, rather than in a direct and discrete form (Rijsdijk 2005b; Neale and Cardon 1992; Arseneault et al. 2003). Additionally, an independent pathway model can be used to test for common genetic and environmental influences directly influencing each of the observed variables, without the need of a higher-order factor. Therefore, our purpose in this study was to apply different analytical models to PSQI data from a representative sample of middle-aged twins of both sexes, with the objective of examining which genetic and environmental structure fits best to the data. With this approach we aimed to: (1) examine the etiology of the associations between the PSQI components; and (2) explore the psychometric structure of the PSQI, testing and comparing different alternative structures for the questionnaire.

## Method

### Participants

The sample was composed of 2129 participants from the Murcia Twin Registry (MTR), coming from 1178 families: 158 monozygotic (MZ) male (120 complete) pairs, 197 dizygotic (DZ) male (171 complete) pairs, 213 MZ female (194 complete) pairs, 218 DZ female (187 complete) pairs and 392 DZ opposite sex (279 complete) pairs. The MTR is a population based twin registry in the Region of Murcia, South East of Spain. Description of the MTR and recruitment processes has been extensively discussed elsewhere (Ordonaña et al. 2013, 2019). The representativeness of the MTR cohort has also been satisfactorily tested in a previous publication (Ordoñana et al. 2018). The sample was 45.5% male (n = 973) and 32.5% MZ (n = 696). The mean age was 53.7 (SD = 7.3; Range 43–71).

All MTR protocols and instruments, as well as data collection procedures and analysis derivatives thereof, have been approved by the Research Ethics Committee of the University of Murcia and meet the legal requirements of confidentiality and protection of personal data. Participants provided written informed consent when interviewed in person or oral consent by telephone interview. Results on univariate analyses of sleep quality components using this sample have been reported previously (Madrid-Valero et al. 2018).

### Measures

#### Zygosity

Zygosity was established using DNA test in 338 pairs of twins. When this was not possible, a 12-item questionnaire was used focusing on the degree of similarity and mistaken identity between twins. This questionnaire-based zygosity has proved to be a reliable and valid measure with an agreement in nearly 96% of cases (Ordonana et al. 2013).

#### Sleep quality—Pittsburgh sleep quality index

Sleep quality was measured using the PSQI, a widely used questionnaire in clinical practice and research for more than 30 years (Buysse et al. 1989). This questionnaire measures sleep quality over the previous month. This instrument has seven sub-scales: (1) subjective sleep quality; (2) sleep latency; (3) sleep duration; (4) habitual sleep efficiency; (5) sleep disturbances; (6) use of sleeping medication and (7) day-time dysfunction. These seven subscales are derived from the 19 questions and are coded in a scale from 0 to 3). Some of the sub-scales are based on a single item (e.g. subjective sleep quality—during the past month, how would you rate your sleep quality overall? With four response options: very good, fairly good, fairly bad, very bad). Other dimensions are composed of several items (e.g. sleep latency or sleep disturbances) and for other components an index must be calculated using information from several questions (e.g. sleep efficiency—number of hours of sleep/number of hours spent in bed × 100). Information about the scoring of the PSQI and full item description can be consulted elsewhere (Buysse et al. 1989). These seven sub-scales yield a global score ranging from 0 to 21. It is important to note that higher scores represent poorer sleep quality. A cut-off has been suggested to distinguish between people with an adequate sleep quality (PSQI global score ≤ 5) and those suffering from poor sleep quality (PSQI global score > 5) (Buysse et al. 1989; Royuela Rico and Macías Fernández 1997). The Spanish validation of this questionnaire was used for this study (Royuela Rico and Macías Fernández 1997). The PSQI has proven to be a reliable measure with adequate psychometric properties (Carpenter and Andrykowski 1998), high correlations with objective measures (Boudebesse et al. 2014) and its single factor scoring has been validated (Raniti et al. 2018). In this study the Cronbach’s Alpha was 0.73.

### Data analysis

#### Twin modelling

PSQI sub-scales were treated as continuous variables when possible (i.e. Sleep duration; Sleep disturbances, Sleep efficiency and Sleep latency). PSQI components which cannot be described continuously (Subjective sleep quality, daytime dysfunction and use of sleep medication) were treated as ordinal variables according to the PSQI scoring (Buysse et al. 1989). Genetic and environmental influences for the PSQI components were already estimated in this sample, using univariate models, and results showed no significant differences when PSQI sub-scales were analyzed as ordinal or continuous variables (Madrid-Valero et al. 2018). Ordinal variables were analyzed using a liability threshold model. To apply variance component genetic models to categorical twin data, it is assumed that the categories reflect an imprecise measurement of an underlying normal distribution of liability, which would have one or more thresholds to discriminate between the categories (Rijsdijk and Sham 2002). Two of the continuous variables were log + 1 transformed to avoid skewness (i.e. sleep latency and sleep disturbances).

Classic twin designs allow us to disentangle the role of genetic and environmental influences in one trait or behavior (Knopik et al. 2017). This is possible making use of the difference between MZ twins (who share 100% of their DNA) and DZ twins (who share on average 50% of their segregating genes). Genetic influences can be decomposed into additive genetic factors (A; the sum of allelic effects across all loci) and non-additive genetic factors (D; the effect of genetic dominance and, possibly, epistasis). Similarly, environmental influences can be decomposed into common shared environmental influences (C; environmental influences that make people from the same family more alike) and non-shared environmental influences (E; environmental influences that make family members less alike) (Knopik et al. 2017; Verweij et al. 2012).

In a classic twin design, it is not possible to estimate C and D using only data from twins reared together. Hence, the selection of an ACE or an ADE model is made based on the pattern of correlations between MZ and DZ twins. Typically, an ACE model is fitted when the DZ correlation is greater than half of the MZ correlation. On the other hand, an ADE model is fitted when the DZ correlation is less than half of the MZ correlation (Neale and Cardon 1992; Verweij et al. 2012).

Assumptions of the twin design (i.e. equal variances and means for MZ and DZ twins as well as for co-twins) as well as univariate estimates were previously reported elsewhere (Madrid-Valero et al. 2018). In this study we focused on different multivariate models. First a multivariate model including all the PSQI subscales was fitted. This model allows us to estimate both individual sources of variance and also sources of covariance among the PSQI components. In other words, we can determine to what extent the latent variables (i.e. A, C/D and E) correlate across any two of these subscales. Using these estimates, bivariate heritability (bivariate shared environmental and bivariate non-shared environmental) can be calculated which inform us about the proportion of the phenotypic correlation explained by A (C or E). Therefore, we can estimate the etiological correlations (i.e. rA, rC/rD and rE) which inform us about the degree of overlap between two traits. These correlations vary from − 1 to 1 where 0 would mean no overlap and 1 or − 1 complete overlap. Nested models were also fitted to test if one (or two) of the variance components could be dropped without significant worsening of the model fit. The fit of different models and submodels was checked using the likelihood-ratio chi-square test and the Akaike’s information criterion (AIC) (Akaike 1987).

In addition, common pathway and independent pathway models were also fitted. The common pathway model posits genetic and environmental factors on a latent variable, which are filtered down to the seven PSQI subscales; what would correspond with the previously reported unifactorial structure of the PSQI. Therefore, the genetic and environmental influences on that latent factor—namely “sleep quality”—can be calculated. In this model, there is also specific paths for the residual genetic and environmental variance of each sub-scale (Rijsdijk 2005b; Neale and Cardon 1992). The independent pathway model allowed us to estimate common genetic and environmental factors influencing the observed variables directly and not via a higher order factor, as compared to the common pathway model (Rijsdijk 2005a). The comparison between these models can inform about whether the structure of the variance shared between components can be represented in a highly coherent manner, hierarchically, or is better represented by a looser interpretation (Neale and Cardon 1992). As an additional analysis, the 3-factor structure proposed by Cole et al. (2006) was also fitted.

All analyses were run in R (R Core Team 2016) using the OpenMx package (Neale et al. 2016). Models were fitted using the direct symmetric approach as this has proven to have several advantages over the other multivariate models such as the correction of error type 1 rate or parameter bias issues (Verhulst et al. 2019). Age and sex were added to the models as covariates.

#### Factor analysis

Finally, several confirmatory factor analyses were fitted in order to confirm the structure of the PSQI in this sample according to the previously suggested models. First, a confirmatory factor analysis was performed following the original structure of the PSQI questionnaire (one factor that comprises the seven sub-scales) (Buysse et al. 1989). In addition, a 3-factor model was also fitted following the structure proposed by Cole et al. (2006). This structure has the following factors: (1) Sleep efficiency (sleep duration and habitual sleep efficiency; (2) Perceived sleep quality (subjective sleep quality, sleep latency and use of sleeping medication; (3) Daily disturbances (sleep disturbances and daytime dysfunction). Analyses were performed using the lavaan package (Rosseel 2012) in R (R Core Team 2016). In order to evaluate model fit, the following statistics were used: *Comparative Fit Index* (CFI), *Tuckey-Lewis Index* (TLI), *Root Mean Square Error of Approximation* (RMSEA), and *Standardized Root Mean-Square Residual* (SRMR). These statistics have been selected following recommendations from previous publications establishing that values close to 0.95 for TLI and CFI, 0.06 for RMSEA and 0.08 for SRMR indicate a good model fit (Hu and Bentler 1999). Analyses were performed using just one twin (randomly selected) from each twin pair (N = 949 observations).

## Results

### Multivariate twin model

Cross twin correlations for the seven components of sleep quality are displayed in Table 1. The best fit for the multivariate model including the 7 PSQI sub-scales as separate factors was provided by an AE model (ADE_AIC_ = 13,793; ACE_AIC_ = 13,793; AE_AIC_ = 13,778, p_ACE-AE_ = 0.05; CE_AIC_ = 18,500, p_ACE-CE_ < 0.001; E_AIC_ = 13,886, p_AE-E_ < 0.001). Heritability estimates from the multivariate model ranged from 0.21 (95% CI 0.12, 0.33) for sleep efficiency to 0.40 (95% CI 0.21, 0.57) for use of sleeping medication (Table2).

**Table 1:** Cross twin correlations

	rMZ (CI 95%)	rDZ (CI 95%)
Subjective sleep quality	0.36 (0.23,0.47)	0.12 (0.02,0.21)
Sleep latency	0.17 (0.06,0.27)	0.15 (0.07,0.23)
Sleep duration	0.25 (0.14,0.35)	0.13 (0.05,0.21)
Habitual sleep efficiency	0.16 (0.05,0.26)	0.16 (0.08,0.23)
Sleep disturbances	0.28 (0.20,0.38)	0.20 (0.11,0.27)
Use of sleeping medication	0.52 (0.34,0.67)	0.13 (− 0.03,0.28)
Daytime dysfunction	0.47 (0.29,0.61)	− 0.01 (− 0.17,0.17)
Global PSQI	0.35 (0.24,0.45)	0.14 (0.05,0.23)

**Table 2:** Additive genetic and nonshared environmental influences on the sleep quality components of the PSQI and their association

	Latency	Duration	Efficiency	Disturbances	Sleep quality	Use of sleeping medication	Daytime dysfunction
Latency	**A = 0.23 (0.14,0.32)** **E = 0.77 (0.68,0.86)**						
Duration	A = 0.34 (0.02,0.56) E = 0.66 (0.44,0.98)	**A = 0.25 (0.13,0.35)** **E = 0.75 (0.65,0.87)**					
Efficiency	A = 0.24 (0.04,0.45) E = 0.76 (0.55,0.96)	A = 0.25 (0.10,0.37) E = 0.75 (0.63,0.90)	**A = 0.21 (0.12,0.33)** **E = 0.79 (0.67,0.88)**				
Disturbances	A = 0.30 (0.12,0.52) E = 0.70 (0.48,0.88)	A = 0.32 (0.12,0.55) E = 0.68 (0.45,0.88)	A = 0.28 (0.10,0.46) E = 0.72 (0.54,0.90)	**A = 0.32 (0.23,0.39)** **E = 0.68 (0.61,0.77)**			
Sleep quality	A = 0.42 (0.28,0.58) E = 0.58 (0.42,0.72)	A = 0.38 (0.22,0.56) E = 0.62 (0.44,0.78)	A = 0.35 (0.16,0.52) E = 0.65 (0.48,0.84)	A = 0.42 (0.27,0.56) E = 0.58 (0.44,0.73)	**A = 0.30 (0.17,0.43)** **E = 0.70 (0.57,0.83)**		
Use of sleeping medication	A = 0.54 (0.20,0.82) E = 0.46 (0.18,0.80)	* *	A = 0.43 (0,0.86) E = 0.57 (0.14,1)	A = 0.74 (0.36,1) E = 0.26 (0,0.64)	A = 0.49 (0.20,0.81) E = 0.51 (0.19,0.80)	**A = 0.40 (0.21,0.57)** **E = 0.60 (0.43,0.79)**	
Daytime Dysfunction	A = 0.84 (0.19,1) E = 0.16 (0,0.81)	A = 0.25 (0,0.69) E = 0.75 (0.31,1)	A = 0.38 (0,0.98) E = 0.62 (0.02,1)	A = 0.52 (0.22,0.77) E = 0.48 (0.23,0.78)	A = 0.39 (0.09,0.63) E = 0.61 (0.37,0.92)	A = 0.51 (0.03,1) E = 0.49 (0,0.97)	**A = 0.36 (0.20,0.55)** **E = 0.64 (0.45,0.80)**

Bold figures represent within-trait standardized components of the variance and figures below the diagonal represent the standardized components of the covariance; *A* additive genetic influence, *E* non-shared environmental influence

*The phenotypic correlation between use of sleeping medication and sleep duration (− 0.06) cannot be expressed as a proportion since there are both negative and positive values. Therefore, this phenotypic correlation is due to − 0.08 to genetic factors and 0.02 to non-shared environmental factors which adds up to − 0.06

With regard to the association between PSQI components, we found phenotypic correlations ranging from − 0.48 (95% CI − 0.52, − 0.44) between subjective sleep quality and sleep duration, to 0.64 (95% CI 0.60, 0.69) between sleep efficiency and sleep duration. The lowest phenotypic correlations were found between sleep duration and use of sleeping medication (rPH − 0.06; 95% CI − 0.12, − 0.01) and sleep latency and daytime dysfunction (rPH 0.15; 95% CI 0.09, 0.21) (Table 3).

**Table 3:** Phenotypic, genetic and non-shared environmental correlations among the PSQI sub-scales

	Latency	Duration	Efficiency	Disturbances	Sleep quality	Use of sleeping medication
Latency						
Duration	**rA = − 0.45 (− 0.69, − 0.04)** **rE = − 0.27 (− 0.37, − 0.18)** **rPH = − 0.31 (− 0.36, − 0.27)**					
Efficiency	**rA = − 0.42 (− 0.65, − 0.09)** **rE = − 0.36 (− 0.45, − 0.28)** **rPH = − 0.38 (− 0.43, − 0.32)**	**rA = 0.69 (0.45,0.84)** **rE = 0.63 (0.56,0.70)** **rPH = 0.64 (0.60,0.69)**				
Disturbances	**rA = 0.36 (0.10,0.55)** **rE = 0.32 (0.25,0.41)** **rPH = 0.33 (0.28,0.36)**	**rA = − 0.35 (− 0.60, − 0.13)** **rE = − 0.30 (− 0.37, − 0.21)** **rPH = − 0.31 (− 0.34, − 0.27)**	**rA = − 0.39 (− 0.59, − 0.16)** **rE = − 0.36 (− 0.43, − 0.27)** **rPH = − 0.36 (− 0.40, − 0.33)**			
Sleep quality	**rA = 0.74 (0.49,0.97)** **rE = 0.36 (0.28,0.43)** **rPH = 0.46 (0.42,0.50)**	**rA = − 0.66 (− 0.87, − 0.46)** **rE = − 0.41 (− 0.49, − 0.31)** **rPH = − 0.48 (− 0.52, − 0.44)**	**rA = − 0.65 (− 0.81, − 0.43)** **rE = − 0.41 (− 0.50, − 0.33)** **rPH = − 0.47 (− 0.51, − 0.43)**	**rA = 0.69 (0.52,0.84)** **rE = 0.43 (0.35,0.51)** **rPH = 0.51 (0.49,0.55)**		
Use of sleeping medication	**rA = 0.48 (0.19,0.79)** **rE = 0.18 (0.06,0.31)** **rPH = 0.27 (0.20,0.32)**	rA = − 0.26 (− 0.58,0.10) rE = 0.03 (− 0.15,0.17) **rPH = − 0.06 (− 0.12, − 0.01)**	rA = − 0.30 (− 0.63,0.21) **rE = − 0.17 (− 0.34, − 0.04)** **rPH = − 0.21 (− 0.27, − 0.15)**	**rA = 0.50 (0.24,0.89)** rE = 0.10 (− 0.12,0.23) **rPH = 0.24 (0.17,0.30)**	**rA = 0.55 (0.20,0.79)** **rE = 0.31 (0.12,0.50)** **rPH = 0.39 (0.33,0.45)**	
Daytime dysfunction	**rA = 0.44 (0.06,0.73)** rE = 0.03 (− 0.09,0.16) **rPH = 0.15 (0.09,0.21)**	rA = − 0.17 (− 0.47,0.29) **rE = − 0.22 (− 0.38, − 0.09)** **rPH = − 0.21 (− 0.27, − 0.13)**	rA = − 0.25 (− 0.67,0.20) **rE = − 0.16 (− 0.29, − 0.01)** **rPH = − 0.18 (− 0.23, − 0.13)**	**rA = 0.49 (0.14,0.73)** **rE = 0.23 (0.10,0.37)** **rPH = 0.32 (0.25,0.37)**	**rA = 0.44 (0.08,0.70)** **rE = 0.34 (0.21,0.47)** **rPH = 0.37 (0.30,0.43)**	**rA = 0.33 (0.02,0.92)** rE = 0.19 (− 0.03,0.38) **rPH = 0.25 (0.16,0.31)**

Bold figures represent significant correlations

*rA* additive genetic correlation, *rE* non-shared environmental correlation, *rPH* phenotypic correlation from the model

As for genetic correlations they were generally high and ranged from − 0.45 (95% CI − 0.69,  − 0.04) between sleep duration and sleep latency, to 0.69 (95% CI 0.45, 0.84) between sleep duration and sleep efficiency. The lowest genetic correlation was found between daytime dysfunction and sleep duration (rA =  − 0.17; 95% CI − 0.47,0.29). We also found significant environmental correlations, although of a lower magnitude. The environmental correlations ranged from − 0.41 (95% CI − 0.49, − 0.31) between subjective sleep quality and sleep duration to 0.63 (95% CI 0.56,0.70) between sleep duration and sleep efficiency. Negligible environmental correlations were found between daytime dysfunction and sleep latency (rE = 0.03; 95% CI − 0.09, 0.16) and between sleep duration and use of sleeping medication (rE = 0.03; 95% CI − 0.15, 0.17) (Table 3).

Most associations among the PSQI components were mainly explained by environmental factors (ranging from 0.76 to 0.51; 15 out of 21 associations). Exceptions where the genetic factors were predominant in the relationship concentrated on use of sleeping medication and its associations with sleep latency (54%; 95% CI 0.20, 0.82), sleep disturbances (74%; 95% CI 0.36, 1); and daytime dysfunction (51%; 95% CI 0.03, 1)]. The association of daytime dysfunction with sleep latency (84%; 95% CI 0.19, 1) and sleep disturbances (52%; 95% CI 0.22, 0.77) was also mainly explained by genetic factors (Table 2).

### Common and independent pathway models

Differences between the multivariate AE model and the AE common and independent pathway models were significant (AIC_AE-multivariate_ = 13,778; AIC_AE-Independent pathway_ model = 13,785; AICAE_-common-pathway-model_ = 14,169; *p* < 0.001), suggesting that neither a general factor of sleep quality nor a model where common genetic and environmental factors influence directly the observed variables capture the relationship between the PSQI subscales better than the seven separate correlating factors. Differences were also significant between the independent pathway model and the common pathway model (p < 0.001). A 3-factor common pathway model, following the structure proposed by Cole et al. (2006) was also fitted but it resulted in a worse fit (AIC = 15,274).

Our common pathway model showed that genetic influences accounted for 32% (95% CI 0.18, 0.43) of variance for the latent factor called sleep quality, while unique environmental influences accounted for the rest [68% (95% CI 0.57, 0.82)]. Regarding the components of sleep quality, common genetic influences explained a substantial proportion of the variance in some sleep quality components [e.g. sleep duration (16%; 95% CI 9%, 24%), sleep efficiency (18%; 95% CI 11%, 26%) and subjective sleep quality (17% 95% CI 9%, 24%)] but not in others [e.g. use of sleeping medication (3% 95% CI 1%, 5%) and daytime dysfunction (4% 95% CI 2%,6%)]. The same applies to environmental influences, up to 39% (95% CI 30%, 49%) of variance was explained by common environmental factors for sleep efficiency and just 6% (95% CI 3%, 10%) for use of sleeping medication. As for variance not explained by the common latent factor, genetic influences ranged from 8% (sleep efficiency and subjective sleep quality) to 32% (95% CI 10%, 45%) (use of sleeping medication). Environmental influences were generally large, ranging from 35% (95% CI 25%, 47%) (sleep efficiency) to 60% (95% CI 52%,67%) (sleep latency) (Supplementary Fig. 1).

The independent pathway model showed a somewhat different landscape. Some components were substantially influenced by common genetic factors (e.g. subjective sleep quality) whereas for others the role of common genetic factors was negligible (e.g. sleep duration and sleep efficiency). Finally, when it comes to specific influences, genetic factors accounted from 0% for subjective sleep quality to 23% (95% CI 8%, 36%) for daytime dysfunction; whereas the effects of environmental factors ranged from 28% (95% CI 18%, 39%) for sleep efficiency to 61% (95% CI 52%, 71%) for sleep latency. (Supplementary Fig. 2).

### Confirmatory factor analysis

Results from the single-factor model provided the following fit statistics: RMSEA = 0.134 (90% CI 0.120, 0.149); SRMR = 0.071; CFI = 0.841; TLI = 0.762; BIC = 15,661.96. Taking into account that sleep duration is used to calculate sleep efficiency and that sleep latency impacts on sleep duration, a model was fitted correlating residuals between sleep duration and efficiency, and between sleep latency and efficiency (Supplementary Fig. 3). This provided much better fit statistics: RMSEA = 0.062 (90% CI 0.046, 0.079); SRMR = 0.033; CFI = 0.971; TLI = 0.949; BIC = 15,479.46. This approach was previously used to analyze the PSQI structure (Raniti et al. 2018). As suggested by Cole et al. (2006) the 3-factor model was also tested. This structure provided good fit statistics: RMSEA = 0.066 (90% CI 0.049, 0.083); SRMR = 0.033; CFI = 0.970; TLI = 0.942; BIC = 15,487.01. However, the unifactorial structure of the PSQI provided the best fit in our sample.

## Discussion

This study aimed to examine the etiological associations between the sleep quality dimensions measured by the PSQI index, as well as the factorial structure of this questionnaire. In doing so, we applied multivariate genetic modelling (1- a multivariate model using the seven components of the PSQI; 2- a common pathway model and; 3- an independent pathway model) to data obtained from a representative sample of middle-aged twins. Our main results highlight several novel and relevant aspects regarding PSQI component architecture: (1) Because of showing the best fit to the data after testing different structural alternatives, the unifactorial structure of the questionnaire is reinforced; (2) A common factor underlying the seven components of the PSQI can be identified, whose estimated heritability (≈30%) equals that of the PSQI global score; (3) Genes and environments influencing the latent factor variance provide different contributions to the specific dimensions of the questionnaire; (4) The extent to which genetic and environmental factors explained each of the associations between paired components also varied. Altogether, this study expands our knowledge about the structure of the PSQI using a genetically informative design.

As with results already reported in this sample using univariate analyses of the PSQI dimensions (Madrid-Valero et al. 2018), results from the 7-variate model showed that these components are substantially influenced by genetic factors, ranging from 19 to 40%; results being largely consistent with findings from previous studies by Genderson et al. (2013), in males, and Barclay et al. (2010a), in young adults. We extended this information by using a common pathway model and an independent pathway model. Our common pathway model showed that a latent factor underlying the seven dimensions of the PSQI is mainly influenced by unique environmental factors (68%) and that shared environmental factors do not have a significant role in explaining differences among the population. The finding of moderate heritability (≈ 30%) is consistent with the variance decomposition analyses of the PSQI global score from previous literature (Madrid-Valero et al. 2018; Genderson et al. 2013; Barclay et al. 2010b) and also with the estimate of two recent meta-analyses of sleep quality which included a variety of indexes for sleep quality other than the PSQI, from single-item questions to objective-physiological measures (Madrid-Valero et al. 2020; Kocevska et al. 2021). While consistency with the PSQI global score was somewhat expected, the coincidence of estimates with meta-analyses comprising different measures represents strong support of the validity of the PSQI as a robust instrument. Interestingly, results from our common pathway model showed that the genetic and environmental influences of the latent factor on the dimensions of ‘daytime dysfunction’ and ‘use of sleeping medication’ are very low, coinciding with results from our confirmatory factor analysis where loadings for these two sub-scales are lowest. This is also reinforced by the independent pathway model estimates, which also support the relative independence of these two components. This could help explaining why neither the common pathway nor the independent pathway models fitted the data better than the multivariate model with 7 separate components.

As for the etiological relationships between dimensions, the comparison with previous studies is difficult since just one offers comparable analyses (Barclay et al. 2010a). Our genetic correlations were somewhat lower than those obtained in the mentioned study; however, its results must be interpreted taking into account the breadth of their reported confidence intervals (from − 1 to 1 in some cases). Moreover, we observed specific differences within such general frame of substantial genetic overlap among the PSQI dimensions. Thus, subjective sleep quality showed strong genetic correlations virtually with all other components (0.44–0.74); or sleep disturbances with sleeping medication and daytime dysfunction (0.49–0.50). However, latency showed moderate genetic correlations with all dimensions (0.36–0.48) but subjective sleep quality (0.74); or duration correlated lower and not significantly with sleeping medication (− 0.26) or daytime dysfunction (− 0.17). This seems especially relevant, since sleep duration is an outcome frequently selected to be explored in molecular genetic approaches (Garfield 2021). Regarding environmental overlap, an even greater variety of results was found. Environmental factors substantially overlapped between some sub-scales (e.g. sleep duration and sleep efficiency or disturbances and sleep quality) while other sub-scales did poorly (e.g. sleep dysfunction and sleep latency or sleep duration and use of sleeping medication). Hence, results from our study showed in general a high genetic overlap between most PSQI components, indicating that the same genetic underpinnings participate in most dimensions. Use of sleep medication and daily dysfunction are the two components that seem to relate differently to the rest, presenting lower and non-significant correlations more frequently. This relative divergence is also apparent for environmental correlations with some of the other components. That would mean that the need of sleeping pills and difficulties during daily activities because of poor sleep present independent characteristics and could be studied separately. These results are reinforced by the independent pathway model where most of the specific genetic factors are low, except those of the two mentioned components.

Our results also showed that, in general, environmental influences were the main variable explaining the association among the sleep quality dimensions, also consistent with the previously cited study. Only in five of the associations did genetic factors explain more than 50% of the phenotypic correlation (latency/use of sleeping medication, latency/daytime dysfunction, sleep disturbances/use of sleeping medication, sleep disturbances/daytime dysfunction, and use of sleeping medication/daytime dysfunction). On the other hand, dimensions like duration or efficiency always showed their associations with the rest of components where largely driven by environmental factors. Finally, other dimensions, such as sleep disturbances showed a mixed pattern, in such a way that its phenotypic correlations with use of sleeping medication and daytime dysfunction were mainly explained by genes, while environment explained largely its phenotypic correlation with latency, duration and efficiency. Furthermore, our common pathway model illustrates more on these differences, showing that daytime dysfunction and use of sleeping medication hold a relatively high influence of specific genetic factors, while efficiency or duration present an opposite pattern.

Altogether, this set of relationships draws a complex picture where some PSQI components show lesser integration and greater specific genetic influences (i.e., use of sleeping medication and daytime disturbances), others show a greater dependency on environmental factors for its development and associations (i.e., duration and efficiency), while latency and sleep disturbances appear to be in an intermediary position. Finally subjective sleep quality permeates all other relationships. Either way, these differences did not lead to an alternative valid structure of the PSQI since its unifactorial character still seems to better fit the data than any other option. The confirmatory factor analysis replicated results from previous studies of which most have found a unifactorial structure (Manzar et al. 2016). The unifactorial model was superior to the 3-factor model proposed by Cole et al. (2006), and neither did it receive strong support when considering the genetic-environmental relationships among questionnaire dimensions.

In this work the etiological associations between PSQI components have been tested using three different approaches for the first time. Results from this study highlight the major role of environmental influences and also the high genetic overlap between components. These results are of interest for both basic research and clinical practice. The high genetic overlap among some of the components of the questionnaire implies that people with a high genetic predisposition will likely show difficulties in most of the questionnaire domains, while some of them (daily dysfunction and use of sleeping pills) will show some specificity. In parallel, our results showed that environmental factors tend to be more specific to each component, what implies that clinically meaningful information should be gathered on a range of environmental factors that could be associated with sleep dimensions independently. Future studies should aim to identify specific genes and environmental factors that contribute to each of these components. Additionally, results from these analyses could also be informative for other studies in different areas, such as the use of polygenic risk scores for prediction of sleep related problems or helping in the design of clinical trials.

This study has several strengths such as the use of a large representative sample of middle-aged twins from Spain which allowed us to estimate the etiological correlations among sleep quality components with improved statistical power compared to the only previous report (i.e., Barclay et al. 2010a) on these grounds. We have also analyzed the structure of the PSQI and the genetic and environmental influences of the sleep quality components using different approaches such as a multivariate genetic twin model, a common pathway model and a confirmatory factor analysis, which provides us with a very broad picture of the structure and the relationship among the sleep quality dimensions according to the PSQI components. However, this study is not free of limitations. First of all, our results should be interpreted in line with the general assumptions and limitations of twin studies (Verweij et al. 2012). Also, the analyses are focused on sleep quality components which are defined according to the PSQI questionnaire, but other definitions are possible. Moreover, our results should also be replicated in samples of different age ranges as well as in different locations since heritability is a population statistic and may vary from one population to another. Additionally, a more detailed and well-powered analysis focused on gender differences could offer some insights that would help to explain the large and consistent discrepancies found between men and women on sleep quality.

## Conclusions

This study explored the genetic and environmental relationships among sleep quality dimensions, showing substantial and variable genetic overlap between them, together with a considerable specificity regarding their genetic-environmental structure. However, most associations among the PSQI components were mainly explained by environmental factors. Results also found that a latent factor common to all the PSQI components can be identified. Such a latent factor is influenced by genetic factors in around one third, a result consistent with previous publications using the global PSQI score.

## Supplementary Information

Below is the link to the electronic supplementary material.

AE common pathway model. Supplementary file1 (PPTX 45 kb).

AE independent pathway model. Supplementary file2 (PPTX 46 kb).

Confirmatory factor analysis. Supplementary file3 (PPTX 45 kb).

## Data Availability

With restrictions
