# Supplementary material for: Phenotypic, Genetic and Environmental Architecture of the Components of Sleep Quality
Source: Behav Genet. 2022 Aug 25;52(4-5):236–45. doi: 10.1007/s10519-022-10111-0 (PMC9463263; doi:10.1007/s10519-022-10111-0)
Supplement: Supplementary file 1 — AE common pathway model. Supplementary file1 (PPTX 45 kb). [file 10519_2022_10111_MOESM1_ESM.pptx]

## Slide 1
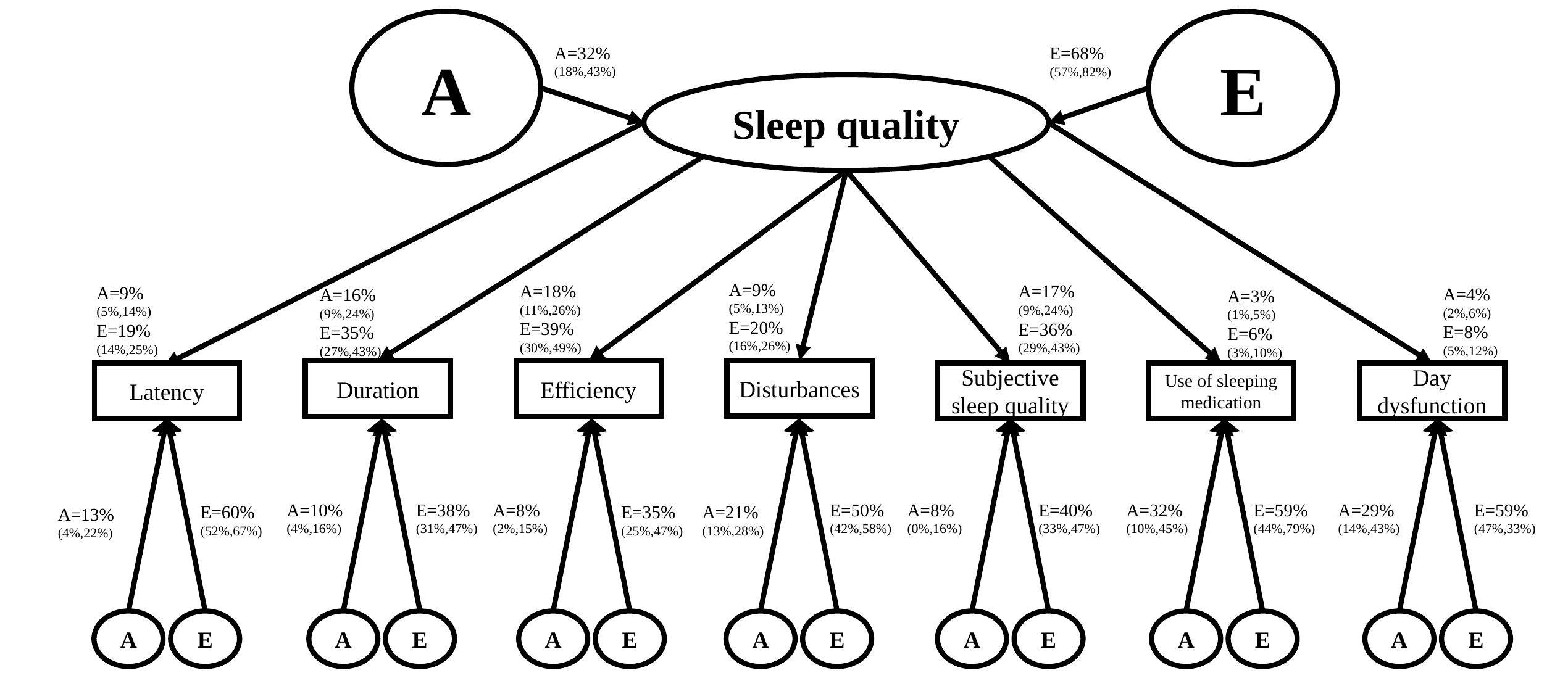

A
E
A=32%
(18%,43%)
E=68%
(57%,82%)
Sleep quality
A=9% (5%,13%)
E=20% (16%,26%)
A=18% (11%,26%)
E=39% (30%,49%)
A=17% (9%,24%)
E=36% (29%,43%)
A=9% (5%,14%)
E=19% (14%,25%)
A=4% (2%,6%)
E=8% (5%,12%)
A=16%
(9%,24%)
E=35%
(27%,43%)
A=3% (1%,5%)
E=6% (3%,10%)
Disturbances
Duration
Efficiency
Latency
Subjective sleep quality
Use of sleeping medication
Day dysfunction
E=38% (31%,47%)
E=50% (42%,58%)
E=40% (33%,47%)
A=32% (10%,45%)
E=59% (44%,79%)
A=29% (14%,43%)
E=59% (47%,33%)
A=10% (4%,16%)
A=8% (2%,15%)
A=8% (0%,16%)
E=60% (52%,67%)
A=21% (13%,28%)
E=35% (25%,47%)
A=13% (4%,22%)
A
E
A
E
A
E
A
E
A
E
A
E
A
E
