# Supplementary material for: Phenotypic, Genetic and Environmental Architecture of the Components of Sleep Quality
Source: Behav Genet. 2022 Aug 25;52(4-5):236–45. doi: 10.1007/s10519-022-10111-0 (PMC9463263; doi:10.1007/s10519-022-10111-0)
Supplement: Supplementary file 2 — AE independent pathway model. Supplementary file2 (PPTX 46 kb). [file 10519_2022_10111_MOESM2_ESM.pptx]

## Slide 1
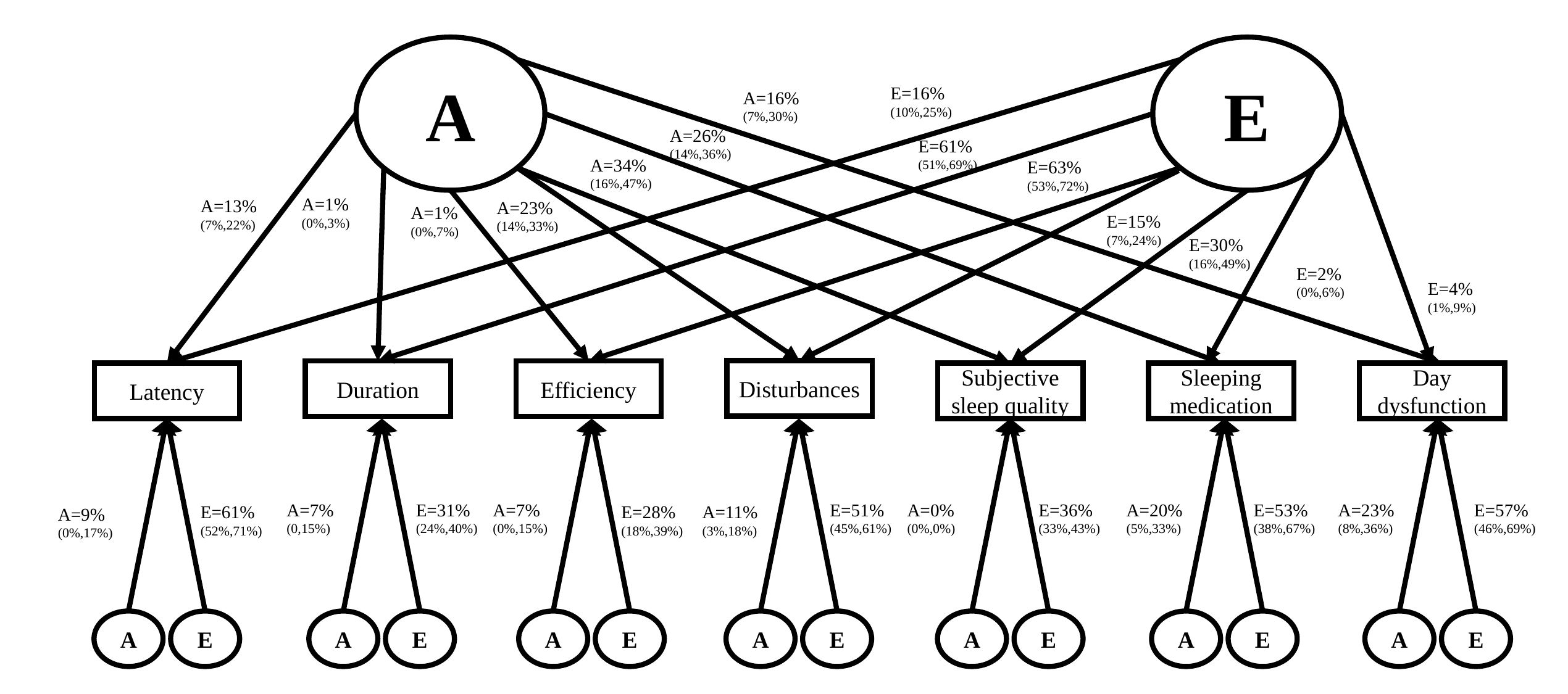

A
E
E=16%
(10%,25%)
A=16%
(7%,30%)
A=26%
(14%,36%)
E=61%
(51%,69%)
A=34%
(16%,47%)
E=63%
(53%,72%)
A=1%
(0%,3%)
A=13%
(7%,22%)
A=23%
(14%,33%)
A=1%
(0%,7%)
E=15%
(7%,24%)
E=30%
(16%,49%)
E=2%
(0%,6%)
E=4%
(1%,9%)
Disturbances
Duration
Efficiency
Latency
Subjective sleep quality
Sleeping medication
Day dysfunction
E=31%
(24%,40%)
E=51%
(45%,61%)
E=36%
(33%,43%)
A=20%
(5%,33%)
E=53%
(38%,67%)
A=23%
(8%,36%)
E=57%
(46%,69%)
A=7%
(0,15%)
A=7%
(0%,15%)
A=0%
(0%,0%)
E=61%
(52%,71%)
A=11%
(3%,18%)
E=28%
(18%,39%)
A=9%
(0%,17%)
A
E
A
E
A
E
A
E
A
E
A
E
A
E
