# Supplementary material for: Phenotypic, Genetic and Environmental Architecture of the Components of Sleep Quality
Source: Behav Genet. 2022 Aug 25;52(4-5):236–45. doi: 10.1007/s10519-022-10111-0 (PMC9463263; doi:10.1007/s10519-022-10111-0)
Supplement: Supplementary file 3 — Confirmatory factor analysis. Supplementary file3 (PPTX 45 kb). [file 10519_2022_10111_MOESM3_ESM.pptx]

## Slide 1
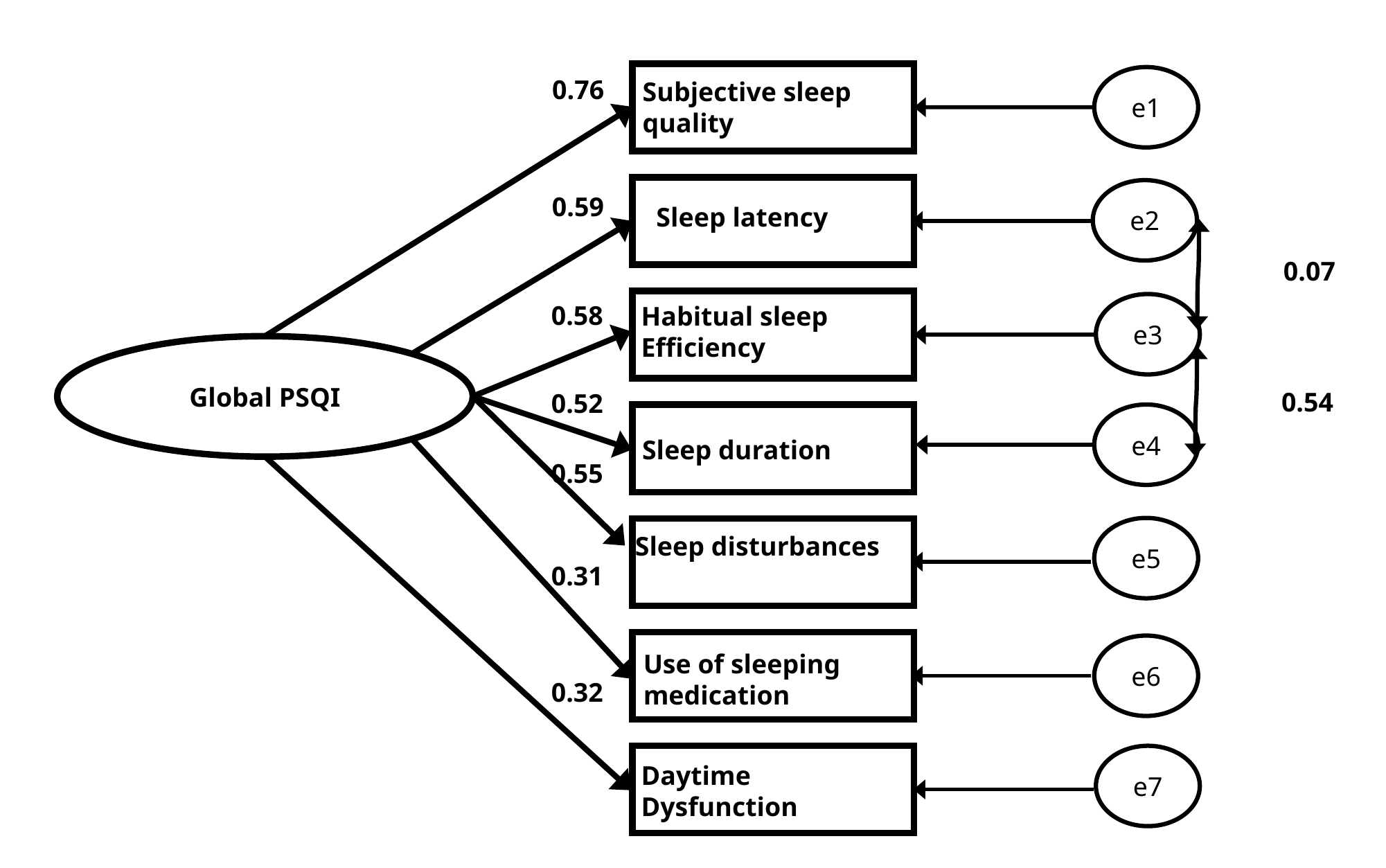

e1
0.76
Subjective sleep quality
e2
0.59
Sleep latency
0.07
e3
0.58
Habitual sleep Efficiency
Global PSQI
0.54
0.52
e4
Sleep duration
0.55
e5
Sleep disturbances
0.31
e6
Use of sleeping medication
0.32
e7
Daytime Dysfunction
